# β-sitosterol isolated from the leaves of* Trema orientalis* (Cannabaceae) promotes viability and proliferation of BF-2 cells

**DOI:** 10.7717/peerj.16774

**Published:** 2024-01-23

**Authors:** Amita Mekarunothai, Markus Bacher, Raveevatoo Buathong, Saraphorn Intarasam, Ngampuk Tayana, Sumet Kongkiatpaiboon, Theppanya Charoenrat, Tiwtawat Napiroon

**Affiliations:** 1 Program in Applied Biological Sciences, Chulabhorn Graduate Institute, Bangkok, Bangkok, Thailand; 2 Department of Biotechnology, Faculty of Science and Technology, Thammasat University, Pathum Thani, Pathum Thani, Thailand; 3 Institute of Chemistry of Renewable Resources, University of Natural Resources and Life Sciences Vienna (BOKU), Tulln an der Donau, Tulln an der Donau, Austria; 4 Department of Biochemistry, Faculty of Medicine Siriraj Hospital, Mahidol University, Bangkok, Bangkok, Thailand; 5 Songkhla Aquatic Animal Health Research and Development Center, Department of Fisheries, Ministry of Agriculture and Cooperatives, Songkhla, Songkhla, Thailand; 6 Drug Discovery and Development Center, Thammasat University, Pathum Thani, Pathum Thani, Thailand; 7 Thammasat University Research Unit in Cannabis and Herbal Products Innovation, Thammasat University, Pathum Thani, Pathum Thani, Thailand

**Keywords:** *Trema orientalis*, Phytosterols, Isolation, Cytotoxicity, Phyochemistry

## Abstract

*Trema orientalis* is a pioneer species in the cannabis family (Cannabaceae) that is widely distributed in Thai community forests and forest edges. The mature leaves are predominantly used as an anti-parasite treatment and feed for local freshwater fish, inspiring investigation of their phytochemical composition and bioactivity. The purpose of this work was to investigate the bioactive compounds in *T. orientalis* leaf extract and their cytotoxicity in the BF-2 fish cell line (ATCC CCL-91). Flash column chromatography was used to produce 25 mL fractions with a mixture solvent system comprised of hexane, diethyl ether, methanol, and acetone. All fractions were profiled with HPLC-DAD (mobile phase methanol:aqueous buffer, 60:40 v/v) and UV detection (wavelengths 256 and 365 nm). After drying, a yellowish powder was isolated from lipophilic leaf extract with a yield of 280 µg/g dry weight. Structure elucidation by nuclear magnetic resonance (NMR) indicated it to consist of pure β-sitosterol. The lipophilic extract and pure compound were evaluated for cytotoxicity using BF-2 cells. MTT assays showed both leaf extract and pure compound at 1 µg/mL to increase cell viability after 24 h treatment. The respective half maximal inhibitory concentration (IC_50_) values of leaf extract and β-sitosterol were 7,027.13 and 86.42 µg/ml, indicating a lack of toxicity in the BF-2 cell line. Hence, *T. orientalis* can serve as a source of non-toxic natural lipophilic compounds that can be useful as bioactive ingredients in supplement feed development.

## Introduction

*Trema orientalis*, a flowering plant in the cannabis family (Cannabaceae), is a common natural pioneer species in tropical forests (Adinortey, Galyuon & Asamoah, 2013; Jin et al., 2020). The placement of *Trema* in Cannabaceae indicates it to be closely related to *Cannabis*; analysis of plastomes has further provided strong supporting evidence that *Trema* and *Cannabis* are sister groups (Yang et al., 2013; Zhang et al., 2018). *T. orientalis* specifically is widely distributed in agricultural areas, disturbed areas, open forests, and forest edges, and has several traditional uses in human disease treatment and as a fish feed supplement, particularly in Thailand and Southeast Asia (HRDI, 2010; Adinortey, Galyuon & Asamoah, 2013; Napiroon et al., 2021). Phytochemical studies of *T. orientalis* have revealed several compounds of interest in different plant parts–tremetol, simiarenol, sterols, and simiarenone in leaves; tremetol, swertianin, scopoletin, fatty acids, and glycosides in stem bark; sterols and fatty acids in roots; and terpenoids, α-amyrin, and cannabinoids in inflorescences (Adinortey, Galyuon & Asamoah, 2013; Parvez et al., 2019; Napiroon et al., 2021). The utilization of *T. orientalis* has been explored *in vitro* and *in vivo*, including its antimicrobial properties (Watt & Breyer-Brandwijk, 1962; Katende, 1995; Napiroon et al., 2021).

Broadly, antimicrobial phytochemicals from medicinal plants are of considerable interest for their utility against infectious microbes, yet some plants are still underused as sources of such agents. *T. orientalis* leaves are indicated to have several activities based on their traditional uses in many localities, such as anti-inflammatory, antibacterial, and antiparasitic activities (Katende, 1995; Watt & Breyer-Brandwijk, 1962). Phytosterols are reported to be commonly present as major metabolites in *T. orientalis* and have a number of pharmaceutical prospects. In veterinary respects, phytosterols are used as an anabolic steroid to promote growth and inhibit *Vibrio* infection (Gallina et al., 2007; Ravi et al., 2020), and some have been reported to stimulate the fertilization of fish and freshwater organisms (Gilman et al., 2003; Leusch & MacLatchy, 2003). Thus, some phytosterols may have significant value in feed supplements and as specialized metabolites for human and animal applications. Accordingly, we examined all reports to date of metabolite and bioassay-guided studies of leaf extracts, which led us to investigate phytosterols from *T. orientalis* and its use as a traditional fish feed ingredient.

Here, we extract phytochemicals from *T. orientalis* leaves and examine the effect of the extract and a pure compound on BF-2 cells, a representative freshwater fish normal cell line. We hypothesized that given its traditional use, *T. orientalis* leaf extract might contain bioactive phytosterols that beneficially affect freshwater fish cells.

The main goals of this study were to purify phytosterols from lipophilic leaf extracts of *T. orientalis* and link potentially active compounds to utilization in freshwater fish feeding by the locals in some areas of Thailand and Southeast Asia. Our study is the new report on the isolation and purification process of phytosterol from *T. orientalis* leaves.

## Materials & Methods

### Plant materials

The leaf materials of *Trema orientalis* were collected from two floristic regions in agricultural areas of northern (Uttaradit province) and northeastern (Bueng Kan province) Thailand. These areas were selected based on notes and field reports on herbarium specimens and other data concerning the use of *T. orientalis* as a local fish feed supplement. Plant samples were identified by using *Trema* key characteristics from Flora of Thailand (Phuphathanaphong, 2015) and another related document. Our voucher specimens (NT011 and NT030) were deposited in the BKF herbarium and indexed in the Index Herbariorum (Thiers, 2020). We selectively collected mature leaves from June to July 2023, during the growing season.

### Extraction procedure

Mature leaves of *T. orientalis* were air-dried at room temperature (30–35 °C) without sunlight or the addition of heat. Dried leaves (300 g) were powdered using an electronic blender, then macerated in methanol with a ratio of 100 g dried materials per 100 mL methanol for 7–10 days in a dark cabinet at room temperature. The extracts were then filtered and concentrated under a vacuum in a water bath at 45–47 °C, 218 mbar pressure to obtain viscous extracts. The obtained viscous extracts were partitioned at a 1:1 ratio between 250 mL of distilled water (hydrophilic phase) and chloroform (lipophilic phase) in a separatory funnel flask. The extract was allowed to settle for 30–60 min in a fume cabinet, after which the lipophilic phase was collected and evaporated to dryness before being stored in methanol for further experiments.

### Compound isolation

The dry leaf lipophilic extract was separated using flash column chromatography (glass column size: 1.7 × 80 cm) with 60 g of silica gel (40–63 µm, Cica Kanto Chemical) as the absorbent. The gradient solvent system consisted of four 100 mL stock solutions of increasing polarity: (1) hexane and diethyl ether mixed in five fractions (95:5, 90:10, 75:25, 50:50 and 0:100%v/v), (2) diethyl ether and methanol mixed in one fraction (75:25%v/v), (3) methanol (100%v/v) and (4) acetone (100%v/v). All obtained 25 mL fractions were kept as sub-fractions and separated on TLC plates with a hexane:ethyl acetate (7:3 v/v) system to observe their chemical profiles; sub-fractions with similar characteristics were subsequently combined. Next, the sub-fractions were evaporated, and the physical properties of the resulting powder or crystals observed before collection in diethyl ether and storage at −20 °C for further HPLC chromatogram and NMR analysis. NMR spectra were recorded at room temperature on a Bruker Avance II 400 (resonance frequencies 400.13 MHz for ^1^H and 100.61 MHz for ^13^C) equipped with a five mm observed broadband probe head (BBFO) with z-gradients. The sample was dissolved in 0.6 ml CDCl_3_ (99.8% D), and chemical shifts were recorded in ppm. For bioassays, a similar sub-fraction was recrystallized with diethyl ether to give pure phytosterols, and then 10 mg of dry pure compound was dissolved in DMSO for bioassay tests.

### HPLC investigation

High-performance liquid chromatography (HPLC) of sub-fractions was performed on a BDS hypersil™ C18 (Thermo Fisher Scientific, Waltham, MA, USA) reverse-phase column (250 × 4.6 mm) using an Agilent 1,100 series with a UV photodiode array detector at wavelengths of 230, 254, and 280 nm. The solvent system included an aqueous buffer consisting of 0.015 M ortho-phosphoric acid (pH 3) and 0.0015 M tetrabutyl ammonium hydroxide as line A and methanol (HPLC analytic grade; Merck, Rahway, NJ, USA) as line B. Sub-fractions were prepared in vials at a concentration of 10 mg/mL in methanol (analytic grade; Merck); only 10 µL was injected for analysis. The solvent flow rate was 1 ml min^−^^1^ over 45 min of chromatogram recording. The mobile phase started at 60% methanol (line B) for 16 min then increased to 90%–100% within 6 min. Finally, 100% methanol was used as a cleanser for 6 min.

### *In vitro* bioassay

The MTT (thiazolyl blue tetrazolium bromide) assay was used to determine cell viability and proliferation *in vitro*. Normally, MTT is a yellowish liquid when dissolved in phosphate buffered saline (PBS). This liquid solution is reduced to an insoluble purple crystal named formazan by mitochondrial reductase enzymes in living cells. The MTT assay method was performed according to the procedure of the American Type Culture Collection (ATCC^®^ 30-1010K, 2011), University Boulevard, USA.

The BF-2 cell line (ATCC CCL-91; Manassas, VA, USA) was stored and cultured at Songkhla Aquatic Animal Health Research and Development Center, Thailand according to the ISO/IEC 17025 standard. Prior to the MTT assay, cells were prepared at a density of 1 × 10^6^ cells/well and cultured at 37 °C with 5% CO_2_ for 24 h. Our experiment tested the final extract and the pure compound at concentrations of 0.1, 1, 10, and 100 µg/ml, and included positive and negative controls. The positive control used medium without extract or pure compound, while the negative control was treated with 0.3% DMSO, the solvent for the extract and pure compound. The amount of DMSO was equivalent to that applied to treated cells. After treatment, cells were incubated at 37 °C with 5% CO_2_ for 24 and 48 h and washed twice with PBS. Then, each treatment was re-suspended in medium and 20 µl of MTT was added. Next, the plates were incubated for 2 h at 37 °C with 5% CO_2_ to allow formazan crystal formation. For harvest, the liquid above the sediment (supernatant) was collected, and DMSO 150 µl was added. The activity was then determined based on absorbance collected *via* a microplate reader (Thermo Scientific Multiskan GO, USA). Percent viability and inhibition concentration (IC_50_) values were calculated using the linear and logarithmic correlation equations. Each experiment was conducted in triplicate and all concentrations were tested in three replications. All statistical analyses used one-way ANOVA with Duncan’s multiple range test. This experimental design aims to screen the morphological changes and toxicity values of BF-2 fish cells that are suitable for freshwater fishes (Babich & Borenfreund, 1985) for further in-depth analysis.

## Results

### Determination of phytosterols in *Trema orientalis* leaf extracts

**Figure 1:**
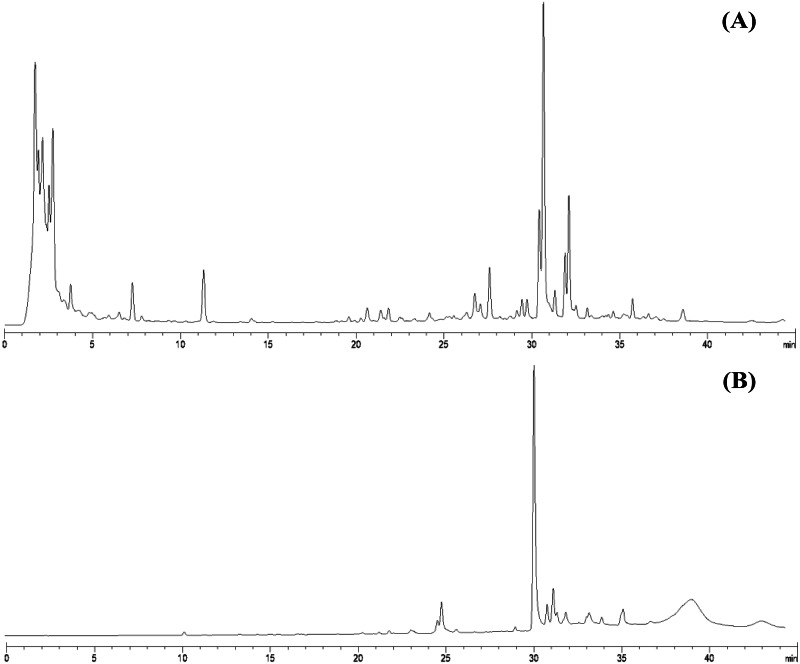
HPLC chromatograms of leaves lipophilic extract (A) and β-sitosterol isolated from leaves lipophilic extracts (B) of *T. orientalis* in 10 mg/mL concentration (mobile phase; aqueous buffer: methanol, 40:60 v/v).

From 30 g dry weight of leaf lipophilic extract, fractionation provided 42 mg of yellowish crystalline powder, which represents a yield of approximately 0.14%. Fractions that exhibited similar TLC chromatograms were combined as one sub-fraction and then qualitative analysis was carried out. Specifically, the fractions combined were fraction 6 from the hexane:diethyl ether (75:25%v/v) mobile phase and fractions 7 and 8 from hexane:diethyl ether (50:50%v/v). Evaporation of this sub-fraction produced a yellowish powder at the bottom of the flask, which was recrystallized in diethyl ether (≥99.9% purity, analytical grade, Merck). The HPLC profiles of the leaf lipophilic extract and the sub-fraction presented similar dominant characteristics between approximate retention times of 30.287 to 30.296 min in the same mobile phase. HPLC chromatograms of both leaf lipophilic extract and sub-fraction are provided in Fig. 1.

### Nuclear magnetic resonance (NMR) structure elucidation

**Figure 2:**
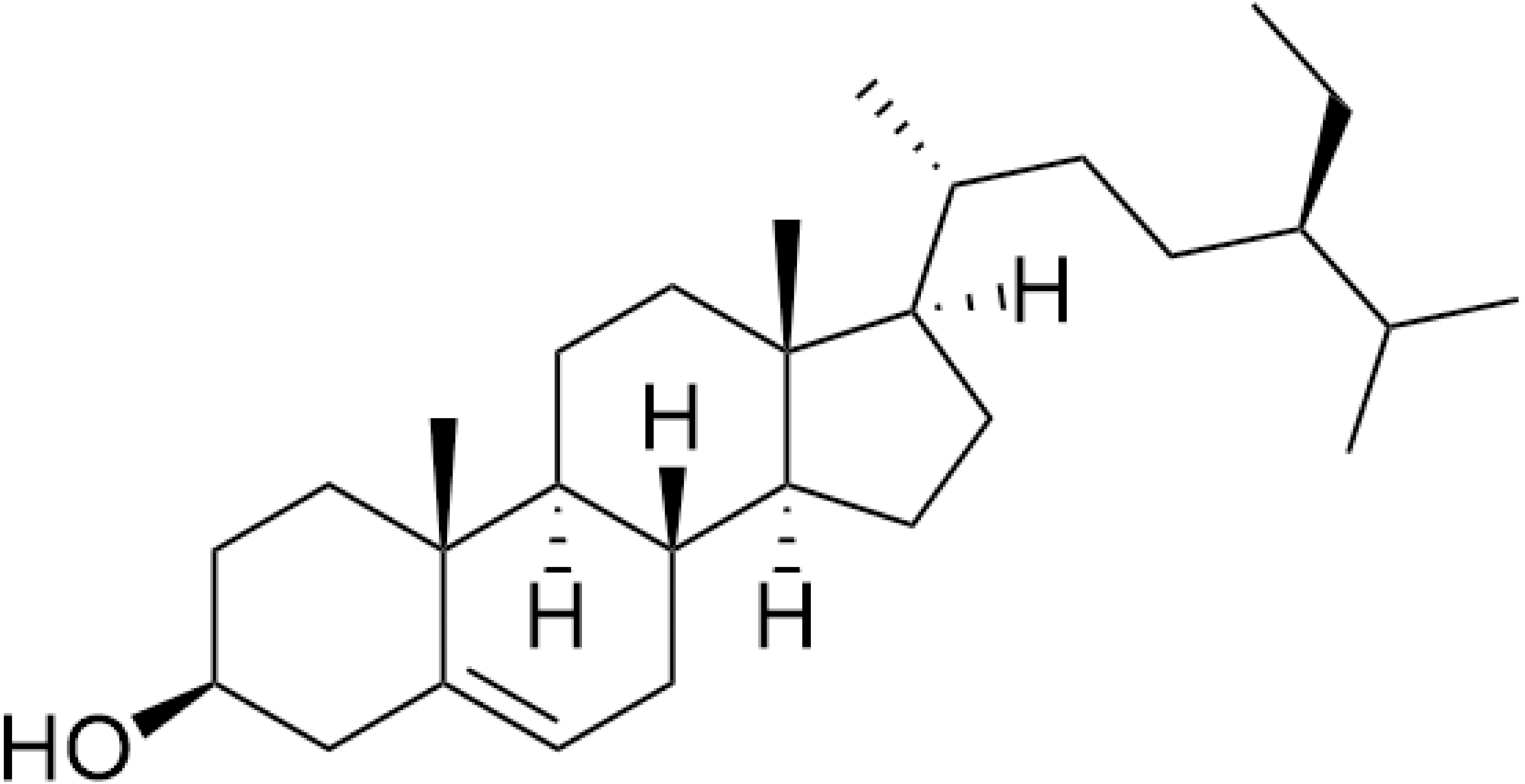
The chemical structure of β-sitosterol (C_29_H_50_O).

The nuclear magnetic resonance (NMR) data are in agreement with those previously reported for β-sitosterol. To the best of our knowledge in *Trema*, β-sitosterol has only isolated from the root bark of this species and the branch of *T. augustifolia*, whereas it was here isolated from leaves part of *T. orientalis*, for the first time. Our NMR data were performed with similar procedures as previously described in Akramov et al. (2020). Specifically, the condition of spectrometer, NMR solvent and standard pulse programs that used for demonstration. The result of 1D (1 H, NOE) and 2D (HSQC) NMR, the yellowish crystalline powder showed the following physical properties indicative of β-sitosterol (C_29_H_50_O): ^1^H-NMR (400 MHz, CDCl_3_, *δ* [ppm], *J* [Hz]): 1.85 (1H, m, H-1a), 1.08 (1H, m, H-1b), 1.84 (2H, m, H-2a, H-16a), 1.51 (1H, m, H-2b), 3.52 (1H, m, H-3), 2.30 (ddd, *J* = 13.1, 5.1, 1.9, H-4a), 2.25 (dm, *J* = 13.1, H-4b), 5.35 (1H, m, H-6), 1.98 (1H, m, H-7a), 1.54 (1H, m, H-7b), 1.46 (1H, m, H-8), 0.93 (2H, m, H-9, H-24), 1.50 (1H, m, H-11a), 1.46 (1H, m, H-11b), 2.01 (1H, m, H-12a), 1.16 (1H, m, H-12b), 1.00 (1H, m, H-14), 1.58 (1H, m, H-15a), 1.07 (1H, m, H-15b), 1.27 (1H, m, H-16b), 1.12 (1H, m, H-17), 0.68 (3H, s, H-18), 1.01 (3H, s, H-19), 1.36 (1H, m, H-20), 0.92 (3H, d, *J* = 6.7, H-21), 1.33 (1H, m, H-22a), 1.02 (1H, m, H-22b), 1.17 (1H, m, H-23), 1.25 (2H, m, H-24^1^), 0.85 (3H, t, *J* = 7.4, H-24^2^), 1.67 (1H, m, H-25), 0.82 (3H, d, *J* = 7.0, H-26), 0.84 (3H, d, *J* = 7.0, H-27). ^13^C-NMR (100 MHz, CDCl_3_, *δ* [ppm]): 37.3 (C-1), 31.7 (C-2), 71.8 (C-3), 42.3 (C-4, C-13), 140.8 (C-5), 121.7 (C-6), 31.9 (C-7), 31.9 (C-8), 50.2 (C-9), 36.5 (C-10), 21.1 (C-11), 39.8 (C-12), 56.8 (C-14), 24.3 (C-15), 28.2 (C-16), 56.1 (C-17), 11.9 (C-18), 19.4 (C-19), 36.2 (C-20), 18.8 (C-21), 34.0 (C-22), 26.1 (C-23), 45.9 (C-24), 23.1 (C-24^1^), 12.0 (C-24^2^), 29.2 (C-25), 19.1 (C-26), 19.8 (C-27). The chemical structure of β-sitosterol (C_29_H_50_O) is depicted in Fig. 2.

### Cytotoxicity experiments

**Figure 3:**
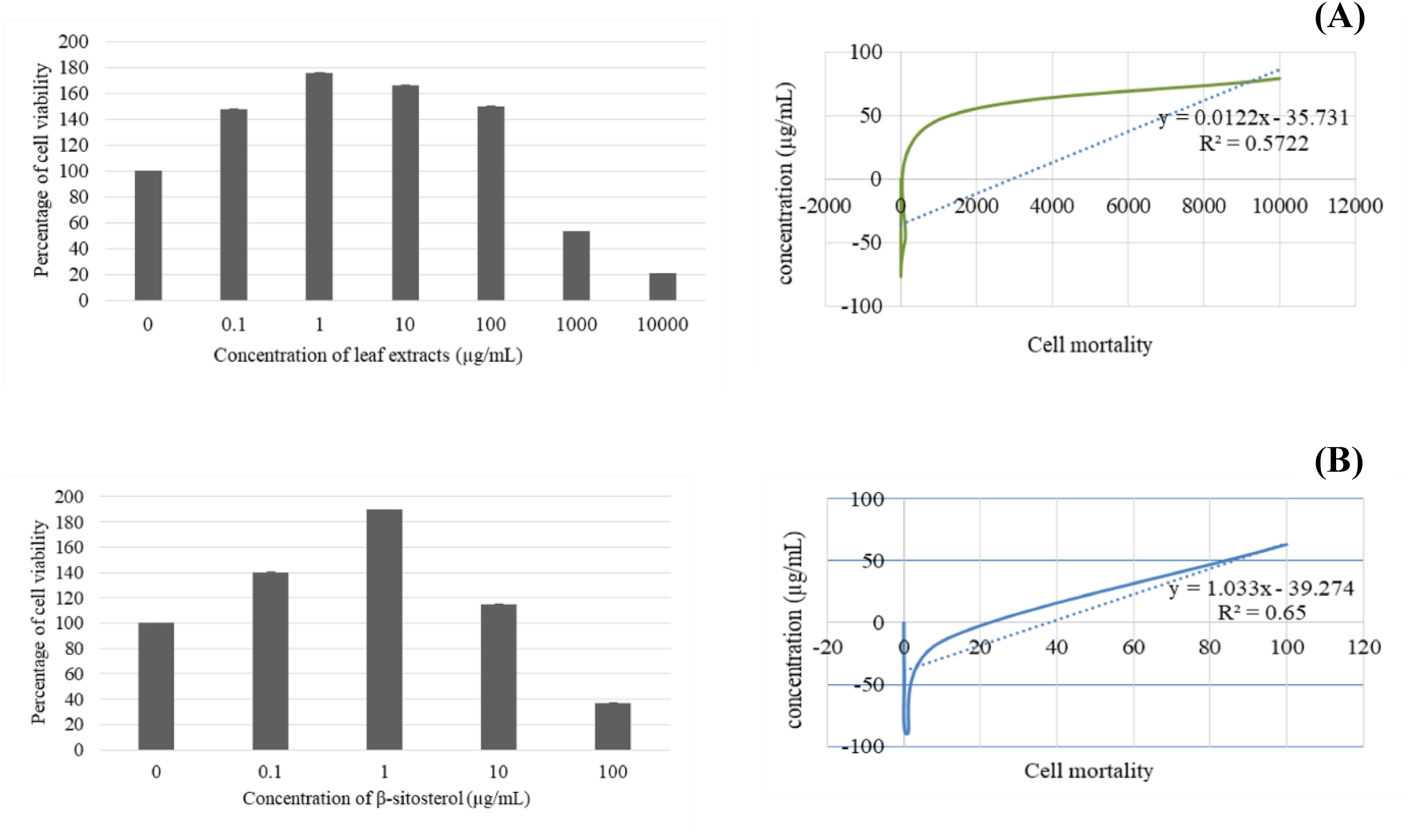
Cytotoxicity and IC_50_ value of leaves lipophilic extract (A) and β-sitosterol isolated from leaves lipophilic extracts (B) of *T. orientalis* after 24 h of cells exposure (MTT assay), Cell viability expressed as the percentage of control (untreated) cell viability. All data expressed as means ± SD (standard deviation) for triplicate independent experiments.

After 24 h, BF-2 cells treated with leaf lipophilic extract at 0.1 and 1 µg/mL exhibited the increased viability, with respective values of 147.93 ± 0.07 and 176.04 ± 0.05, while treatment with 10, 100, 1,000 and 10,000 µg/mL produced lower values of 166.54 ± 0.08, 150.18 ± 0.06, 53.34 ± 0.01, and 20.87 ± 0.004, respectively. Notably, viability percentages greater than the untreated control were observed at concentrations of up to 100 µg/mL. Meanwhile, treatment with β-sitosterol resulted in increased % cell viability at concentrations of 0.1, 1, and 10 µg/mL, with respective values of 140.19 ±  0.06, 189.66 ± 0.11, and 114.88 ± 0.06 µg/mL; treatment with 100 µg/mL resulted in reduced cell viability when compared with the untreated control (Fig. 3).

**Figure 4:**
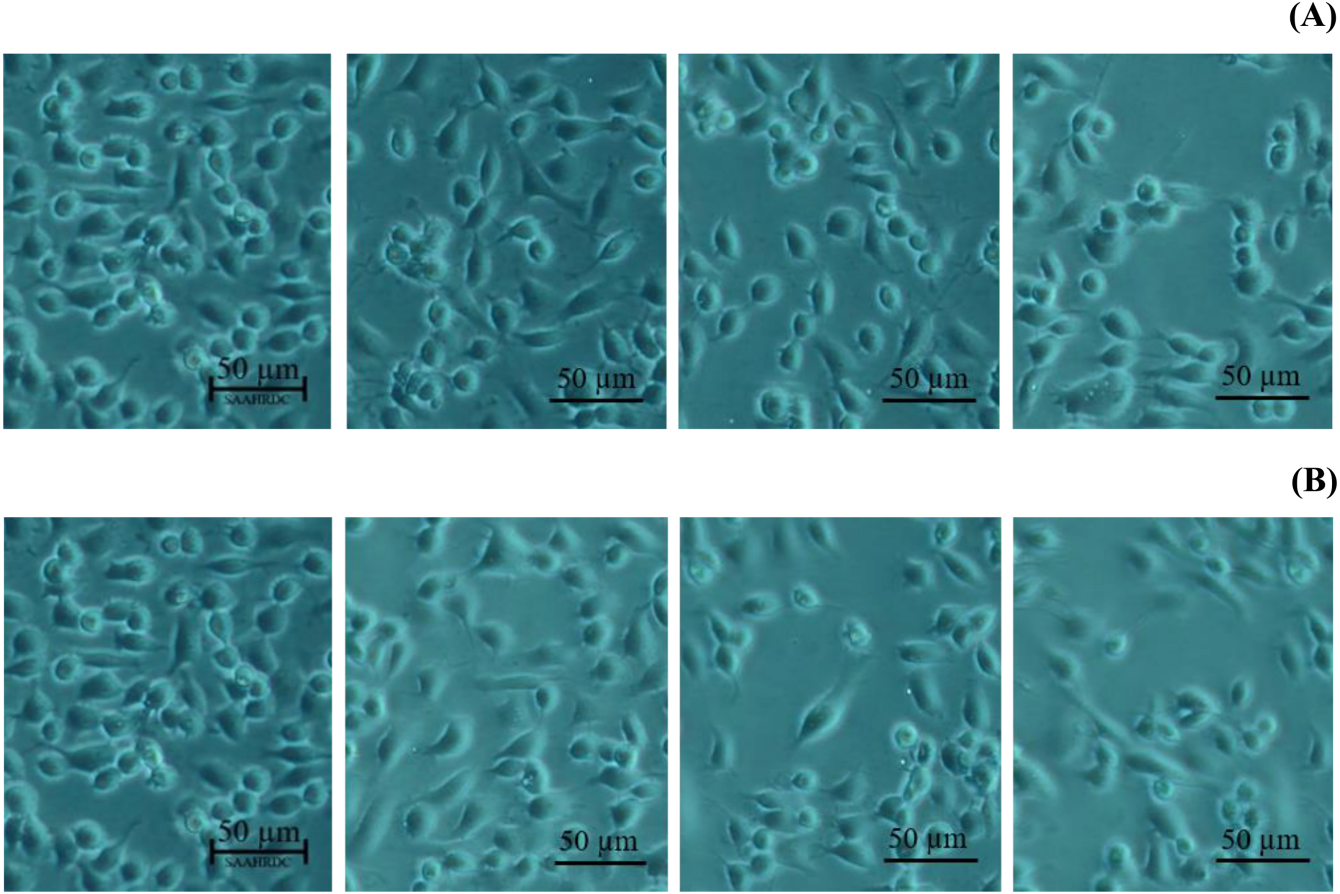
Morphological characters of BF-2 cell lines after 24 h at the highest percentage of cell viability (≥100%) caused by (A) leaves lipophilic extract and (B) β-sitosterol.

**Figure 5:**
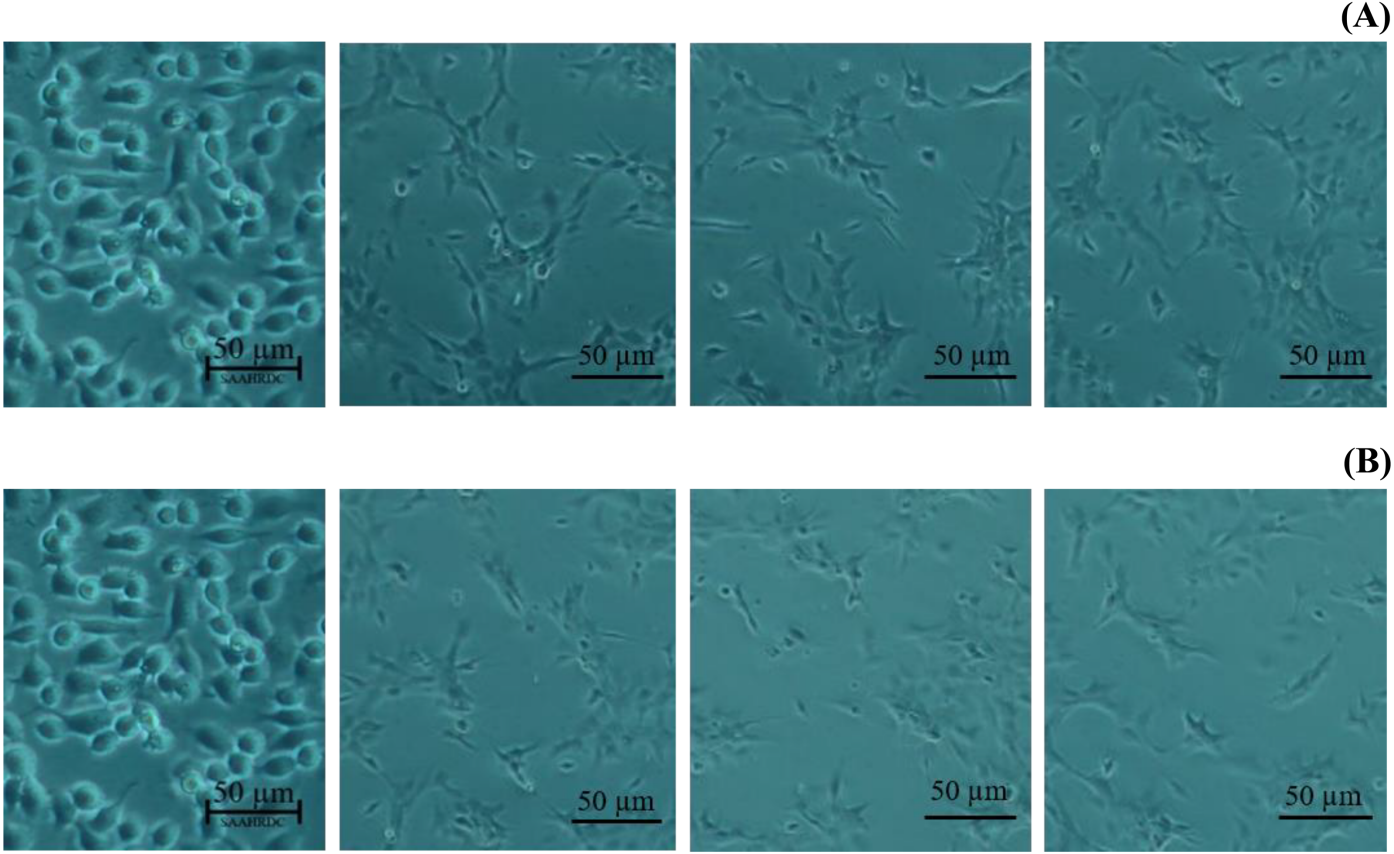
Morphological changes of BF-2 cell lines in the highest concentration of leaves lipophilic extract (A) and β-sitosterol (B) (% cell viability < 100%).

Next, the IC_50_ values of leaf lipophilic extract and β-sitosterol were determined using the criteria of Ballantyne (1999). An IC_50_ of 7,027.13 µg/mL was calculated for leaf lipophilic extract from the equation *y* = −0.0122 ln(*x*)−35.731 (*R*^2^ = 0.5722), and a value of 86.42 µg/mL for β-sitosterol from the equation *y* = 1.033 ln(*x*)−39.274 (*R*^2^ = 0.65); plots are shown in Fig. 3. Representative images showing the morphological characteristics of cells exhibiting increased viability are illustrated in Fig. 4, and corresponding images of cells with reduced viability in Fig. 5. Our results after 24 h indicate these treatments to be potentially nontoxic when used in a concentration less than the respective IC_50_ value, at which a high degree of cell viability inhibition was observed.

## Discussion

### Extraction and isolation

**Figure 6:**
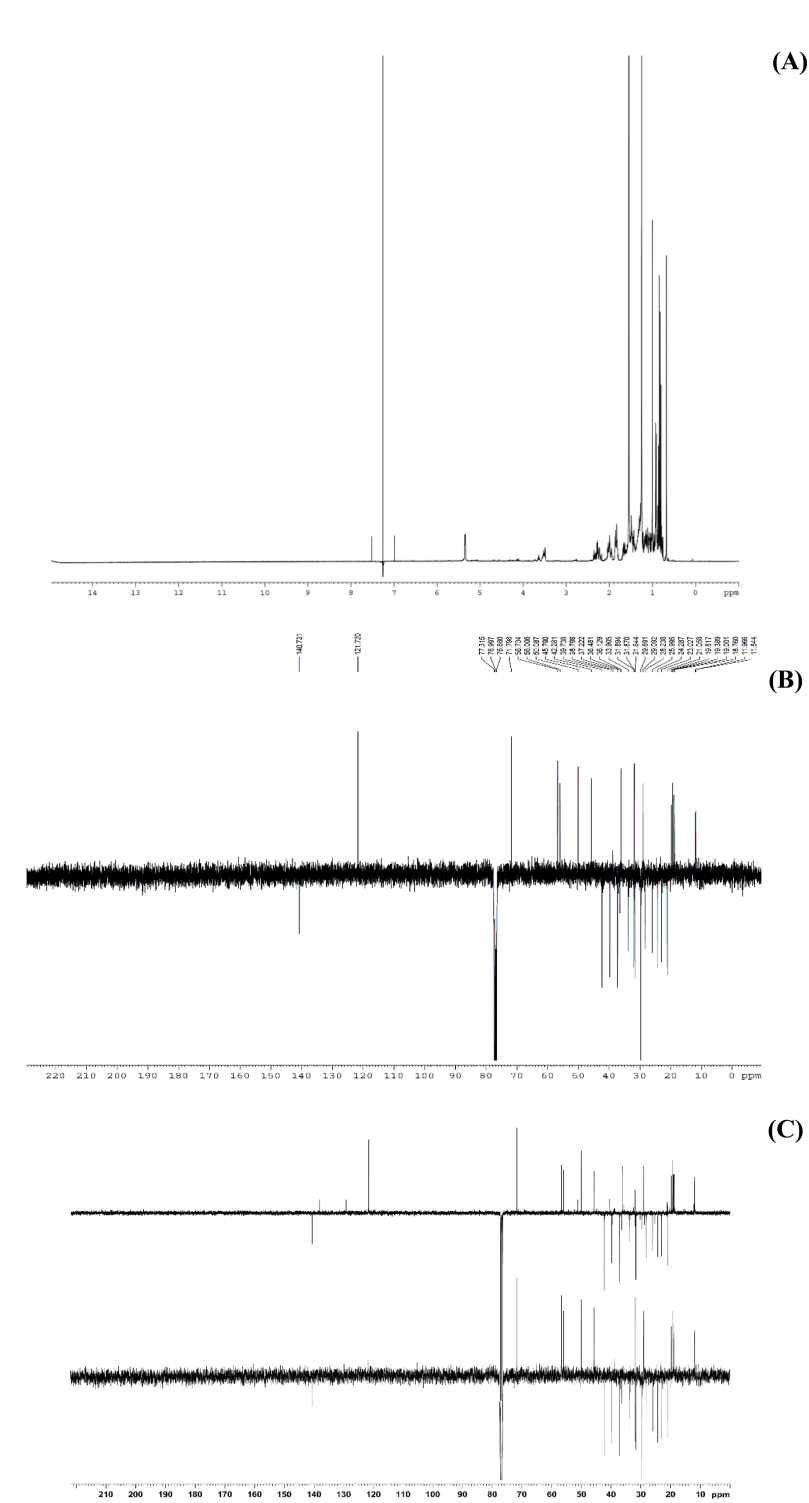
The NMR spectrum evidences of β-sitosterol isolated from *T. orientalis* leaves lipophilic extract, (A) ^1^H NMR spectrum, (B) ^13^C NMR spectrum and (C) the DEPT-135 spectrum.

This study performed bio-guided fractionation using chromatography to isolate active phytosterols of *Trema orientalis* lipophilic leaf extract, from which was acquired β-sitosterol. According to its physical properties, β-sitosterol has a melting point (m.p.) of 136–140 °C and appears as a yellowish crystalline powder, which can be investigated by TLC using the solvent system hexane:ethyl acetate (8:2). Our solvent system ratio developed for β-sitosterol detection is similar to those used in previous literature (Eswaraiah & Elumalai, 2011). HPLC chromatography of the leaf lipophilic extract and β-sitosterol showed a dominant peak at 30.287 min, similar to β-sitosterol chromatograms from the previous reports of Vaidya, Pradhan & Shinde (2017) and Wang et al. (2019). NMR spectra likewise exhibited chemical shift values very close to those reported in the literature for β-sitosterol (Patra et al., 2010; Eswaraiah & Elumalai, 2011; Ododo, Choudhury & Dekebo, 2016). Based on ^1^H NMR and ^13^C NMR, the molecular formula of the isolated compound was determined to be C_29_H_50_O. Fifty H atoms were identified in the ^1^H NMR spectrum: six methyl groups (CH_3_), eleven methylene groups (CH_2_), nine CH bonds, and one hydroxyl group (OH) (Fig. 6A). The appearance of singlets at *δ* 0.68 and 1.01 confirmed the presence of two CH_3_ groups attached to quaternary carbons. The complex multiplets present at *δ* 2.25 and 2.30 revealed that the two CH_2_ are contiguous with the carbon attached to the OH group. The multiplet at *δ* 3.52 is due to a proton connected to the carbon that is attached to the OH group. The signal that appeared for CH at *δ* 5.35 only indicated the olefinic proton.

**Figure 7:**
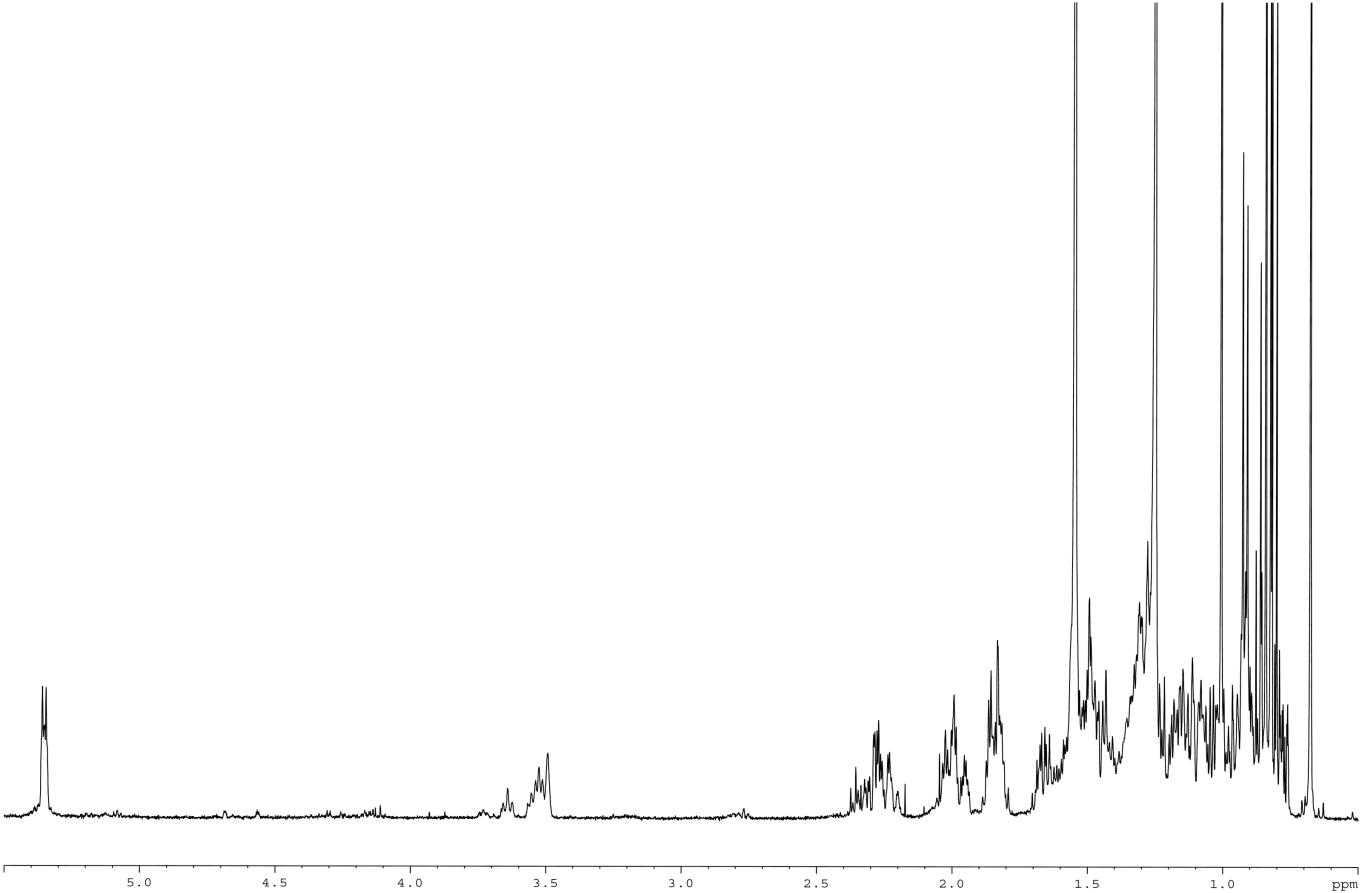
^1^H NMR spectrum of β-sitosterol (400 MHz, CDCl_3_, 0–6 ppm).

Meanwhile, the ^13^C NMR spectrum detected 29 carbons (Fig. 6B). The DEPT-135 spectrum was obtained to classify carbons as CH_3_, CH_2_, CH, or quaternary carbon (QC) (Fig. 6C), and indicated the presence of 26 carbons: six peaks up due to CH_3_ groups, nine peaks up for CH groups, and peaks down indicating the presence of eleven CH_2_ groups. In the ^13^C NMR spectrum, the recognizable signals at 140.80 and 121.70 were assigned to double bonds between carbon atoms, in positions 5 and 6 (C5 = C6) respectively. The signal at *δ* 71.80 was assigned to the C3 β-OH group, and the signals at *δ* 11.90 and 19.40 to angular methyl carbons for C19 and C18, respectively. In addition, the pure compound was confirmed *via* the high-quality ^1^H NMR spectrum (0–6 ppm) that include important signals of methyls (0.8–1.25), 3-H (3.5 (1H, m)), H-5 (5.2 (1H, m)) and O-H (5.0 (H, m)), respectively (Fig. 7). These evidences conform with the previous report of Eswaraiah and Elumalai in 2011 that intensively provided the NMR spectrum of β-sitosterol.

### Cytotoxicity investigation

The viability of BF-2 cells has been taken as an indicator in previous reports examining the toxicity of substances and heavy metals in an aquatic environment (Srikanth et al., 2018; Poornavaishnavi et al., 2019; Małaczewska, Kaczorek-Łukowska & Kazuń, 2021). Fish cell lines are considered to be better predictors for chemical toxicity towards aquatic organisms than mammalian cells, and the BF-2 cell line in particular has been proven a sensitive indicator for toxicological evaluations (Poornavaishnavi et al., 2019). Surprisingly, we observed both *T. orientalis* leaf lipophilic extract and β-sitosterol to promote BF-2 cell proliferation at low concentrations, achieving the greatest viability at 1 µg/mL. The IC_50_ values were correspondingly high, with the extract at 7,027.13 µg/mL and β-sitosterol a lower value of 86.42 µg/mL. Normally, the BF-2 cell line is sensitive and visibly shows toxic effects when subject to low concentrations of substances and toxic metals (Srikanth et al., 2018; Poornavaishnavi et al., 2019). As our results indicated only very high concentrations to have a mortality effect on BF-2 cells for both the extract and the pure compound, this indicates both to likely be nontoxic toward normal cells. Notably, the leaf lipophilic extract contained a mixture of other active compounds alongside β-sitosterol, and synergistic effects could lead to a mixture being more effective than any single constituent (Rasoanaivo et al., 2011); furthermore, synergistic effects may act as bioactivities as well as promote cell proliferation (Gilbert & Alves, 2003).

β-sitosterol is a natural specialized metabolite found in various plant parts (Sen et al., 2012). It has been proven a safe, non-toxic, effective nutritional supplement and has potential health benefits in diverse applications, including against hepatocellular cancer, as an antibacterial, against *Vibrio* infection, and as a growth promoter (Gallina et al., 2007; Ravi et al., 2020; Sen et al., 2012; Vo et al., 2020). Our findings suggest that the presence of β-sitosterol in the leaf lipophilic extract of *T. orientalis* might contribute to its potent promotion of growth in the BF-2 cell line, which represents a sensitive normal cell indicator. Previous experimental studies have shown β-sitosterol to affect the growth of bacterial and human tumor cells (Joy et al., 2012; Sen et al., 2012; Bao et al., 2022). Our findings are also similar to a previous report that found phytosterols in general to act as growth-inducing agents on animal cells (Gallina et al., 2007). Thus, *T. orientalis* leaf lipophilic extract that contains β-sitosterol can be considered a natural phytosterol, which is important for promotion of normal cell growth in freshwater fish in the context of the leaves’ traditional use as a feed ingredient.

## Conclusions

Lipophilic extracts of *T. orientalis* leaves and the β-sitosterol isolated from them (0.14% leaf dry weight) significantly promote cell viability and proliferation in a similar manner, with morphological changes evident in BF-2 cells after treatment with both extract and pure compound. A high IC_50_ value of 7,027.13 µg/mL was obtained for the leaf extract; the presence of nontoxic and beneficial essential metabolites could explain the benefit of the plant in feeding freshwater fish. The IC_50_ value of β-sitosterol was lower but still high, supporting cell viability and proliferation. In addition, the analytical methods used in this study demonstrated reproducible compound patterns that can be used to identify β-sitosterol Continuing study to detect β-sitosterol in other *Trema* may identify other materials for use in natural fish feed ingredients. Our work might also lead to the introduction of *T. orientalis* in agricultural areas as a model source of fish growth promoter agents in the future.

## Supplemental Information

10.7717/peerj.16774/supp-1 Supplemental Information 1 The NMR spectrum evidences of β-sitosterol isolated from *T. orientalis* leaves lipophilic extract

10.7717/peerj.16774/supp-2 Supplemental Information 2 Raw data of percentage of cell viability and IC50 value calculation

## Additional Information and Declarations

### Data Availability

The following information was supplied regarding data availability: The raw data are available in the Supplemental Files.
