# Supplementary material for: β-sitosterol isolated from the leaves of Trema orientalis (Cannabaceae) promotes viability and proliferation of BF-2 cells
Source: PeerJ. 2024 Jan 23;12:e16774. doi: 10.7717/peerj.16774 (PMC10812590; doi:10.7717/peerj.16774)
Supplement: Supplemental Information 1 [file peerj-12-16774-s001.pdf]

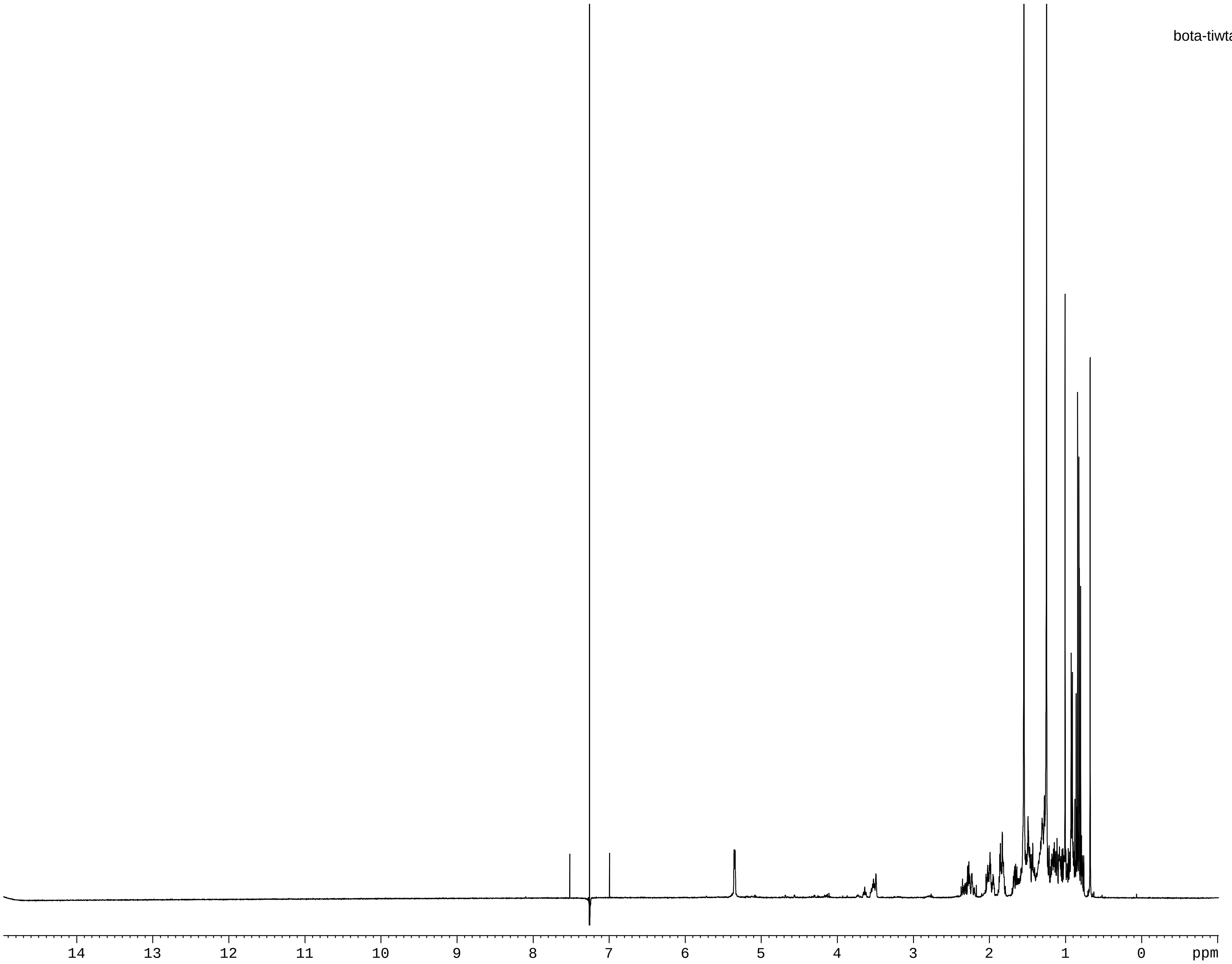

bota-tiwtawat-01, 18 mg

Current Data Parameters
NAME bota-tiwtawat-01
EXPNO 1
PROCNO 1

F2 - Acquisition Parameters
Date_ 20191113
Time 17.05
INSTRUM spect
PROBHD 5 mm CPPBBO BB
PULPROG zg30
TD 32768
SOLVENT CDCl3
NS 144
DS 0
SWH 6393.862 Hz
FIDRES 0.195125 Hz
AQ 2.5624576 sec
RG 114
DW 78.200 usec
DE 10.00 usec
TE 299.1 K
D1 1.00000000 sec
TD0 1

======== CHANNEL f1 ========
SFO1 400.1328009 MHz
NUC1 1H
P1 12.00 usec
PLW1 9.00000000 W

F2 - Processing parameters
SI 32768
SF 400.1300099 MHz
WDW GM
SSB 0
LB -0.30 Hz
GB 0.3
PC 1.00

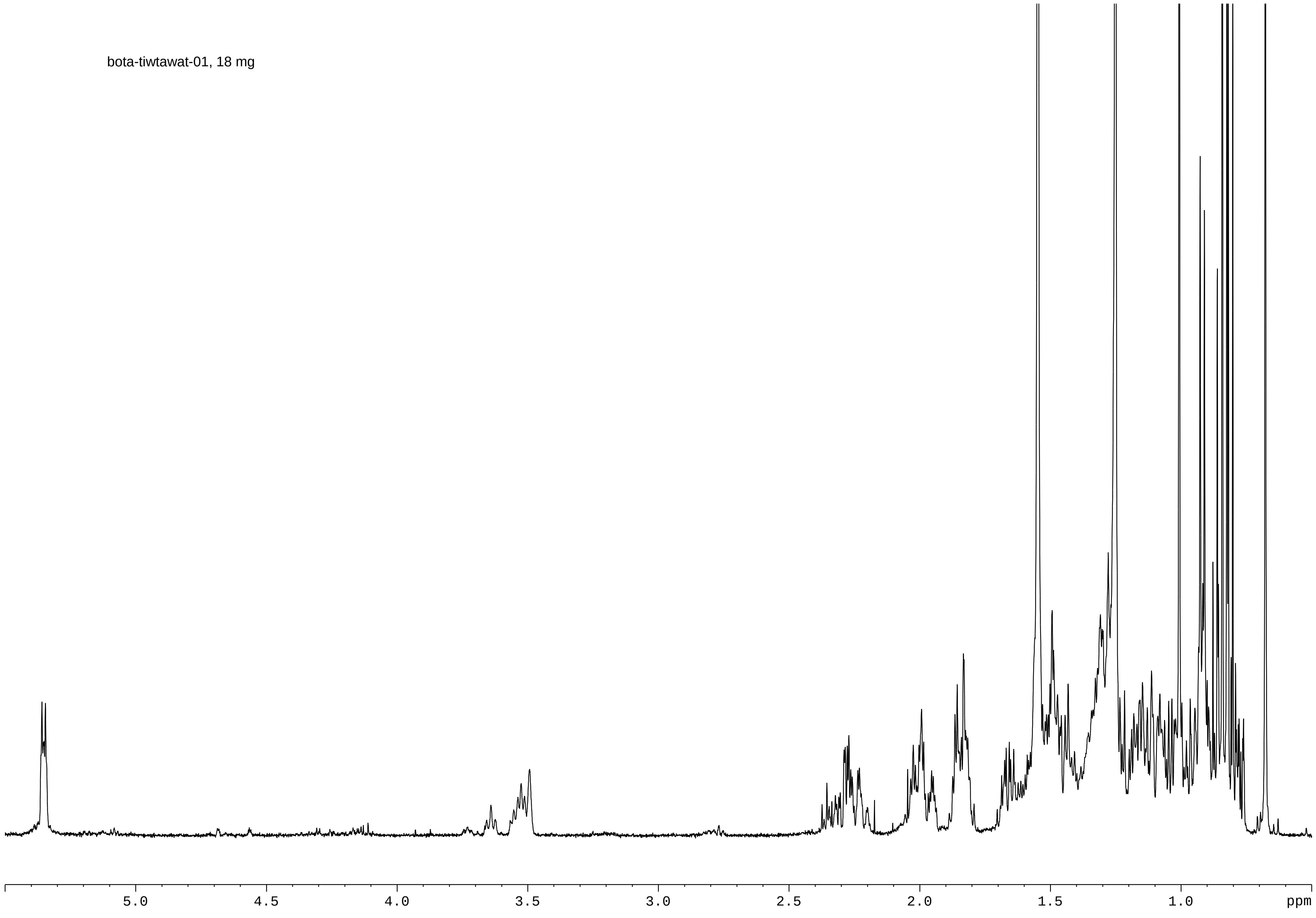

bota-tiwtawat-01, 18 mg
5.0
4.5
4.0
3.5
3.0
2.5
2.0
1.5
1.0
ppm

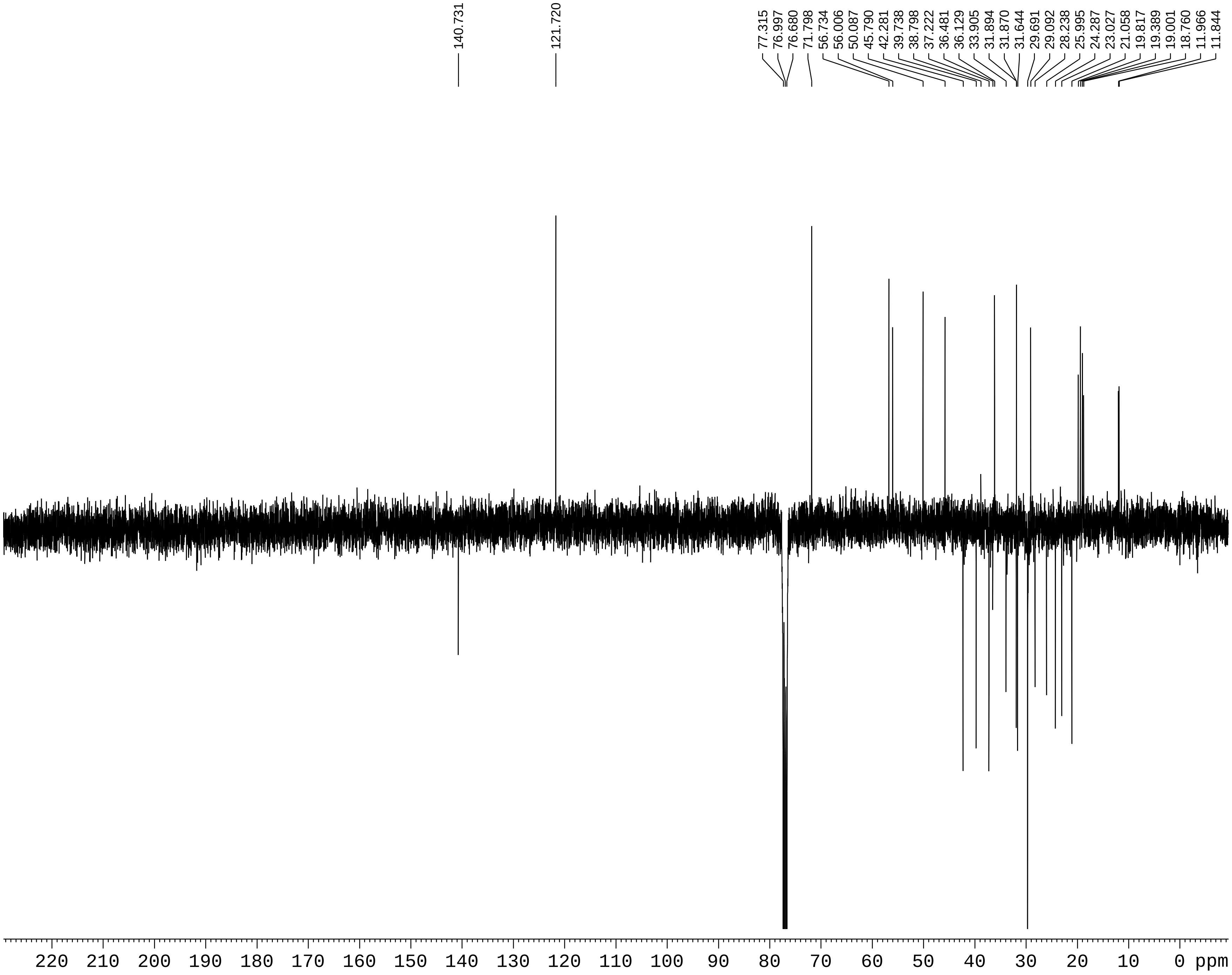

bota-tiwtawat-01, 18 mg (??)
C-13 jmod

```
Current Data Parameters
NAME     bota-tiwtawat-01
EXPNO                   5
PROCNO                  1

F2 - Acquisition Parameters
Date_          20191114
Time              10.23
INSTRUM           spect
PROBHD   5 mm CPPBBO BB
PULPROG            jmod
TD                65536
SOLVENT           CDCl3
NS                10500
DS                    0
SWH           24038.461 Hz
FIDRES         0.366798 Hz
AQ            1.3631488 sec
RG                 2050
DW               20.800 usec
DE                18.00 usec
TE                299.4 K
CNST2       145.0000000
CNST11        1.0000000
D1           2.00000000 sec
D20          0.00689655 sec
TD0                   1

======== CHANNEL f1 ========
SFO1        100.6238364 MHz
NUC1                13C
P1                10.80 usec
P2                21.60 usec
PLW1        32.00000000 W

======== CHANNEL f2 ========
SFO2        400.1320007 MHz
NUC2                 1H
CPDPRG[2        waltz16
PCPD2             80.00 usec
PLW2         9.00000000 W
PLW12        0.20250000 W

F2 - Processing parameters
SI                32768
SF          100.6127719 MHz
WDW                  EM
SSB      0
LB                 1.00 Hz
GB       0
PC                 1.40
```

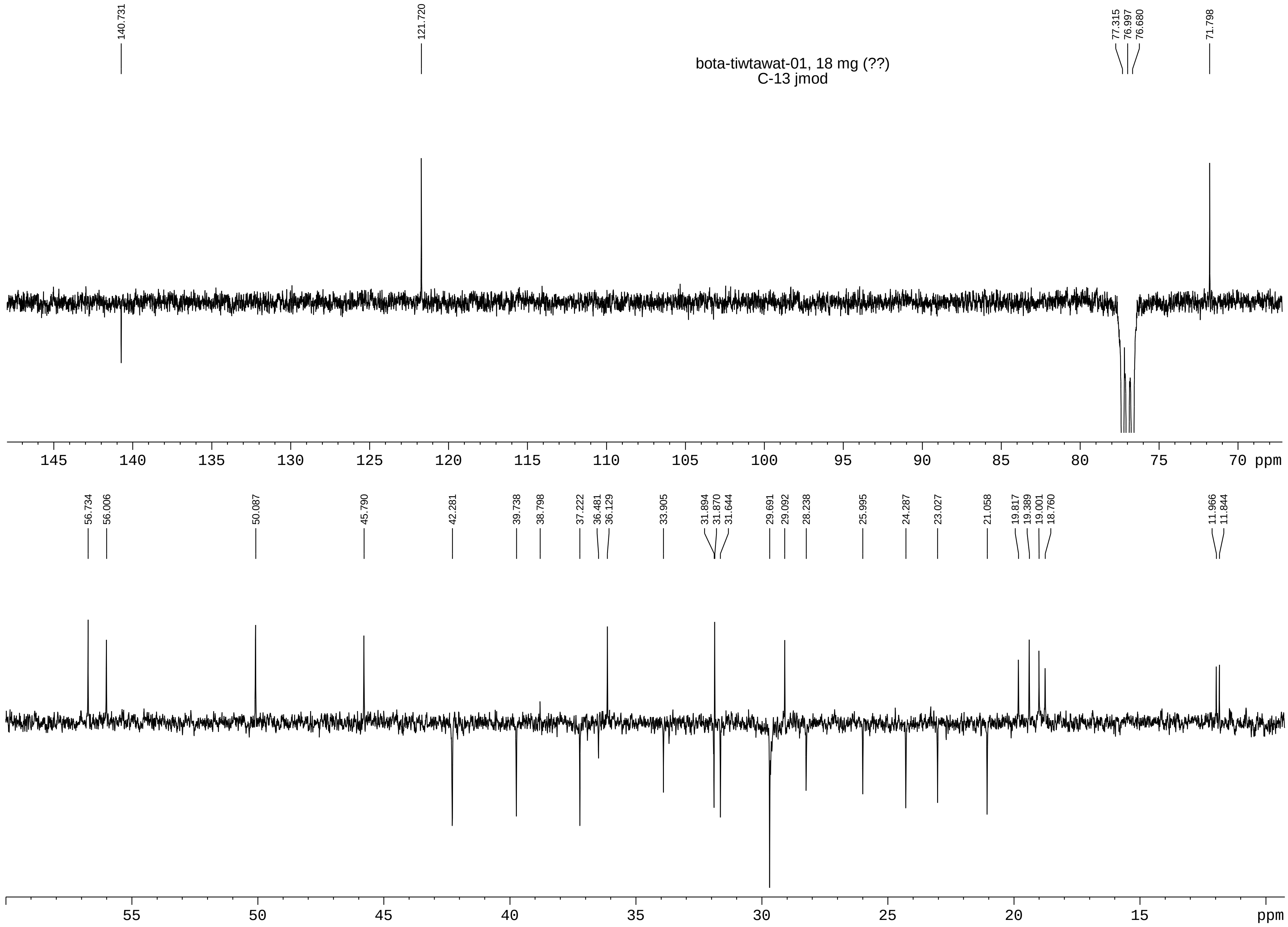
bota-tiwtawat-01, 18 mg (??)
C-13 jmod
140.731
121.720
77.315
76.997
76.680
71.798
145
140
135
130
125
120
115
110
105
100
95
90
85
80
75
70 ppm
56.734
56.006
50.087
45.790
42.281
39.738
38.798
37.222
36.481
36.129
33.905
31.894
31.870
31.644
29.691
29.092
28.238
25.995
24.287
23.027
21.058
19.817
19.389
19.001
18.760
11.966
11.844
55
50
45
40
35
30
25
20
15
ppm

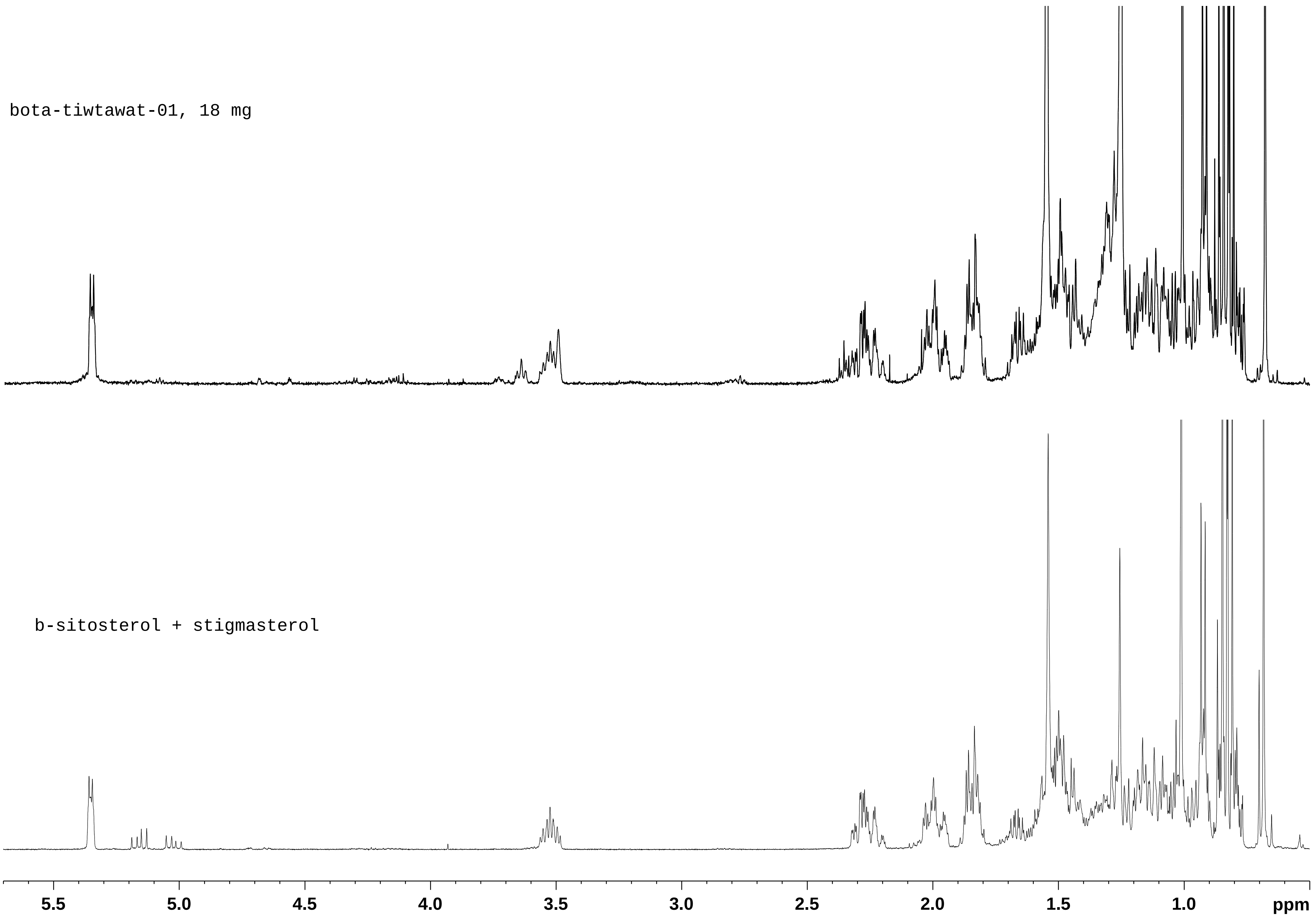
bota-tiwtawat-01, 18 mg
b-sitosterol + stigmasterol
5.5
5.0
4.5
4.0
3.5
3.0
2.5
2.0
1.5
1.0
ppm

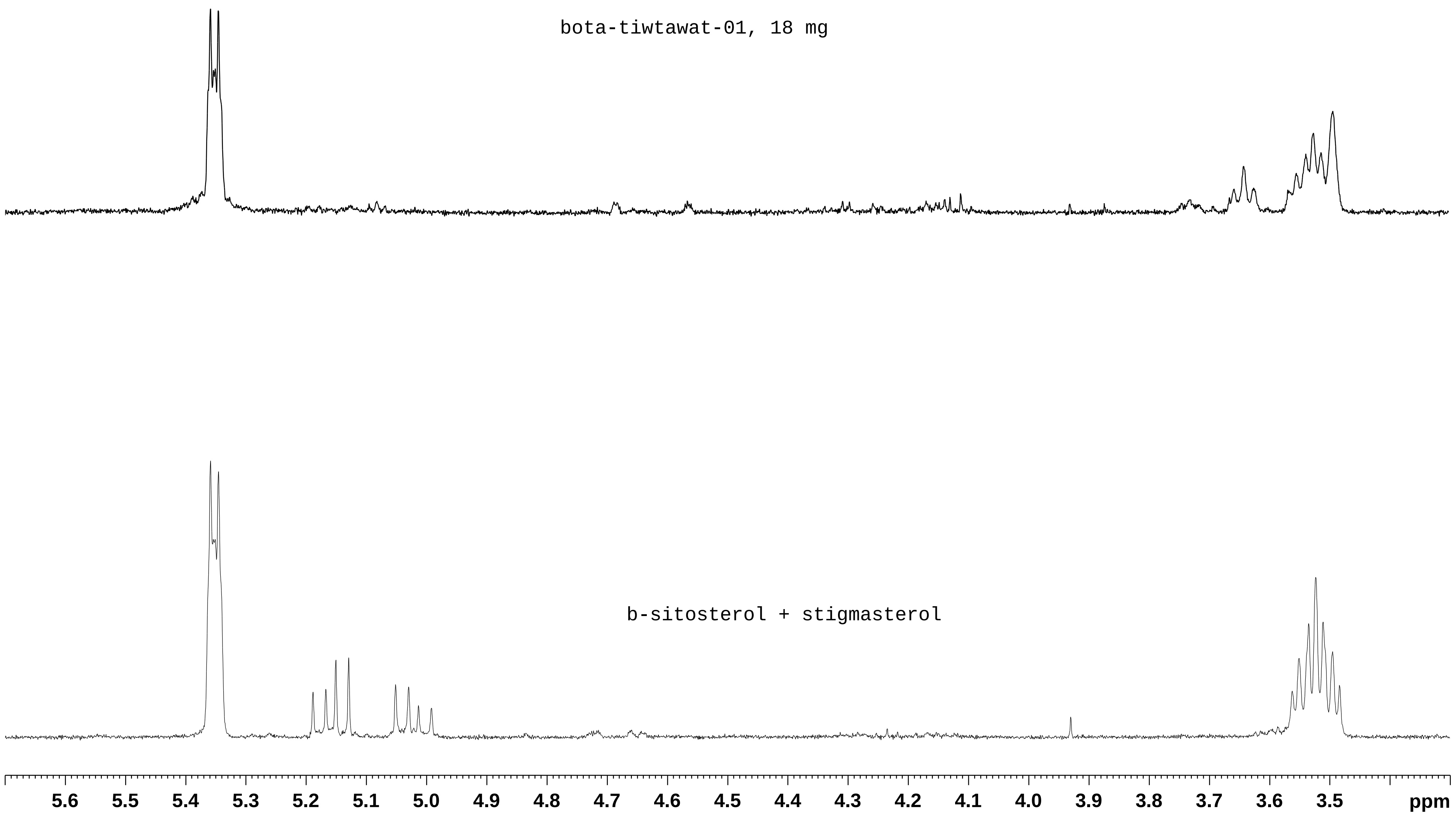
bota-tiwtawat-01, 18 mg
b-sitosterol + stigmasterol
5.6 5.5 5.4 5.3 5.2 5.1 5.0 4.9 4.8 4.7 4.6 4.5 4.4 4.3 4.2 4.1 4.0 3.9 3.8 3.7 3.6 3.5
ppm

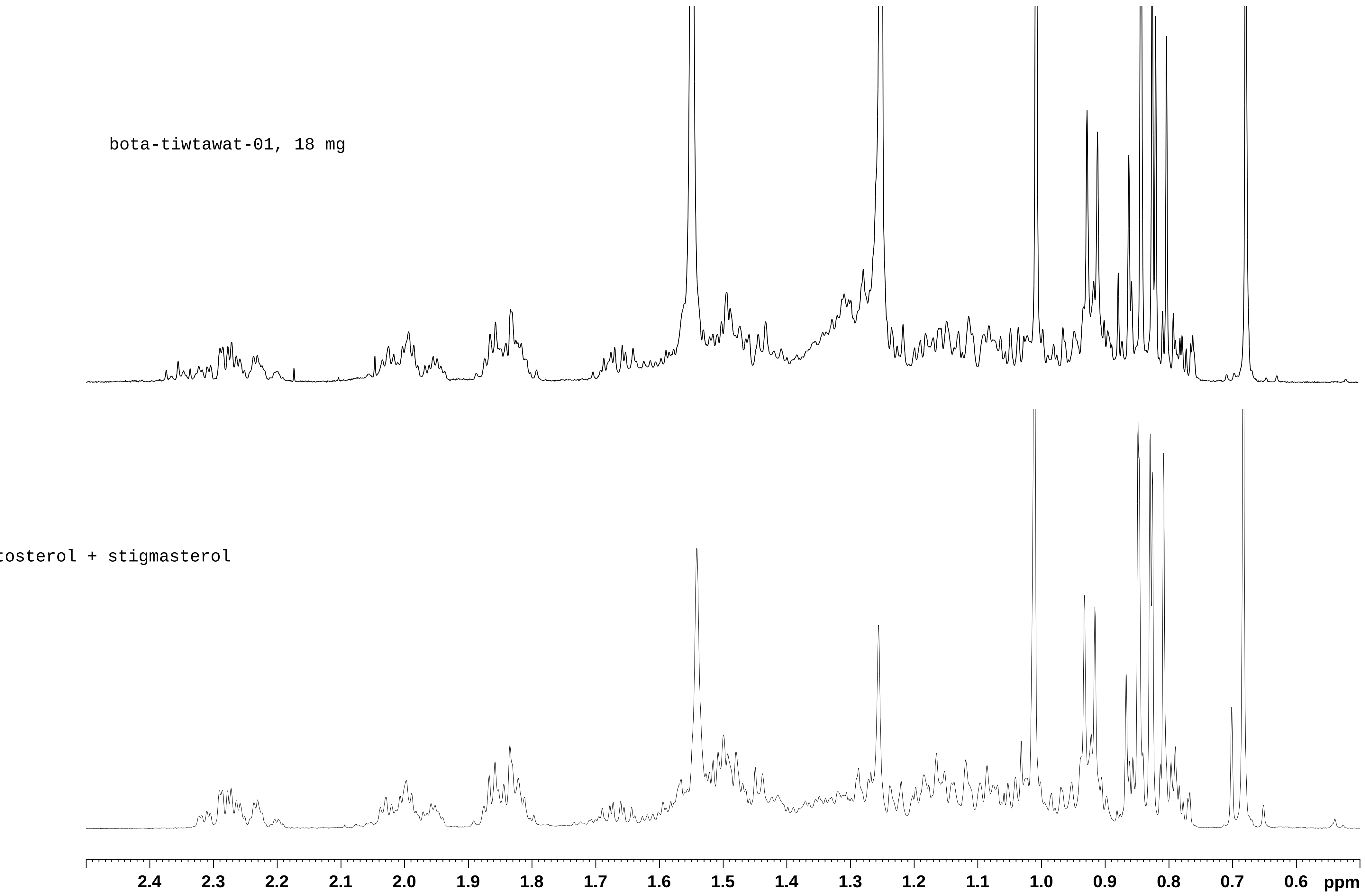

bota-tiwtawat-01, 18 mg
tosterol + stigmasterol
2.4
2.3
2.2
2.1
2.0
1.9
1.8
1.7
1.6
1.5
1.4
1.3
1.2
1.1
1.0
0.9
0.8
0.7
0.6
ppm

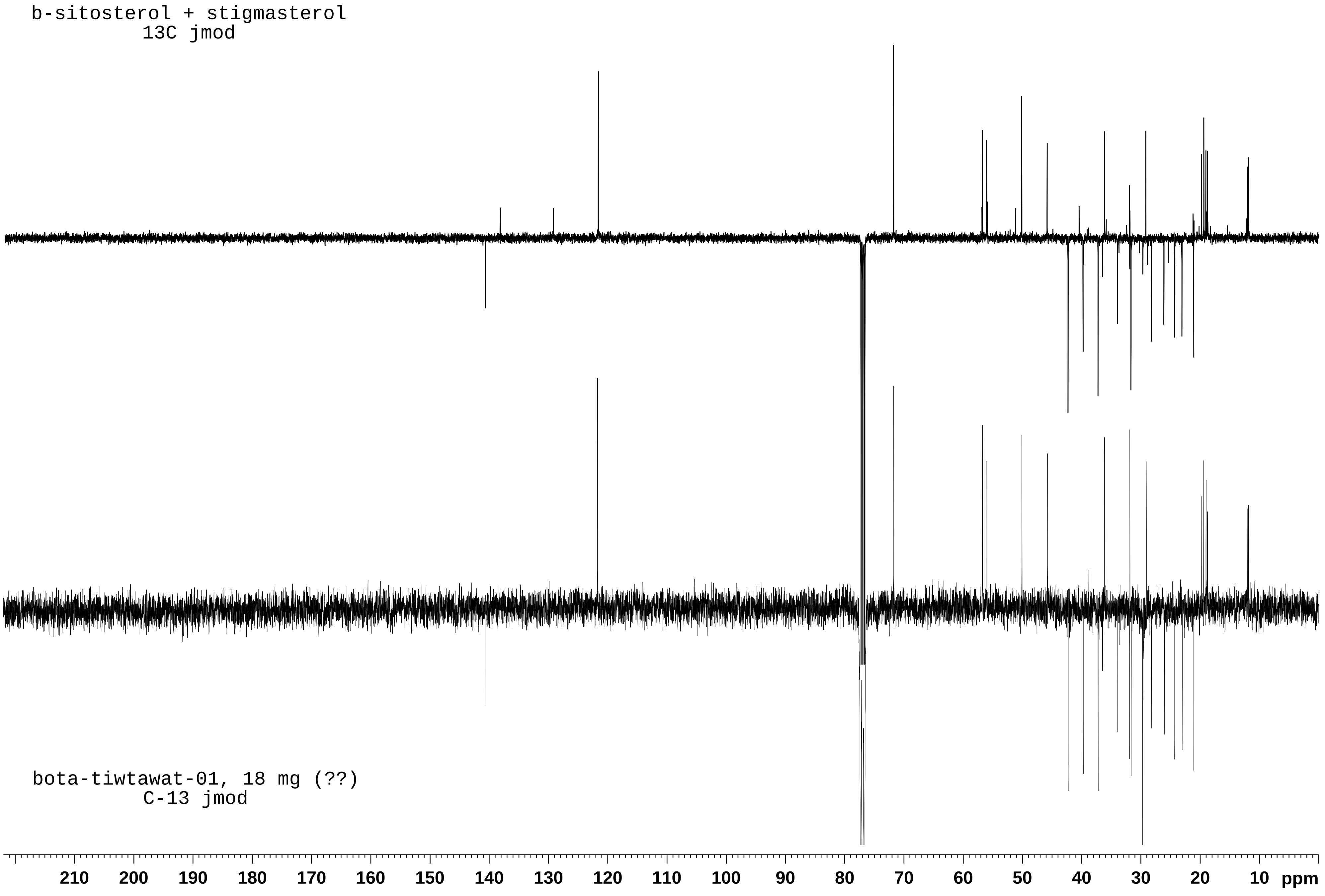
b-sitosterol + stigmasterol
13C jmod
bota-tiwtawat-01, 18 mg (??)
C-13 jmod
210 200 190 180 170 160 150 140 130 120 110 100 90 80 70 60 50 40 30 20 10 ppm

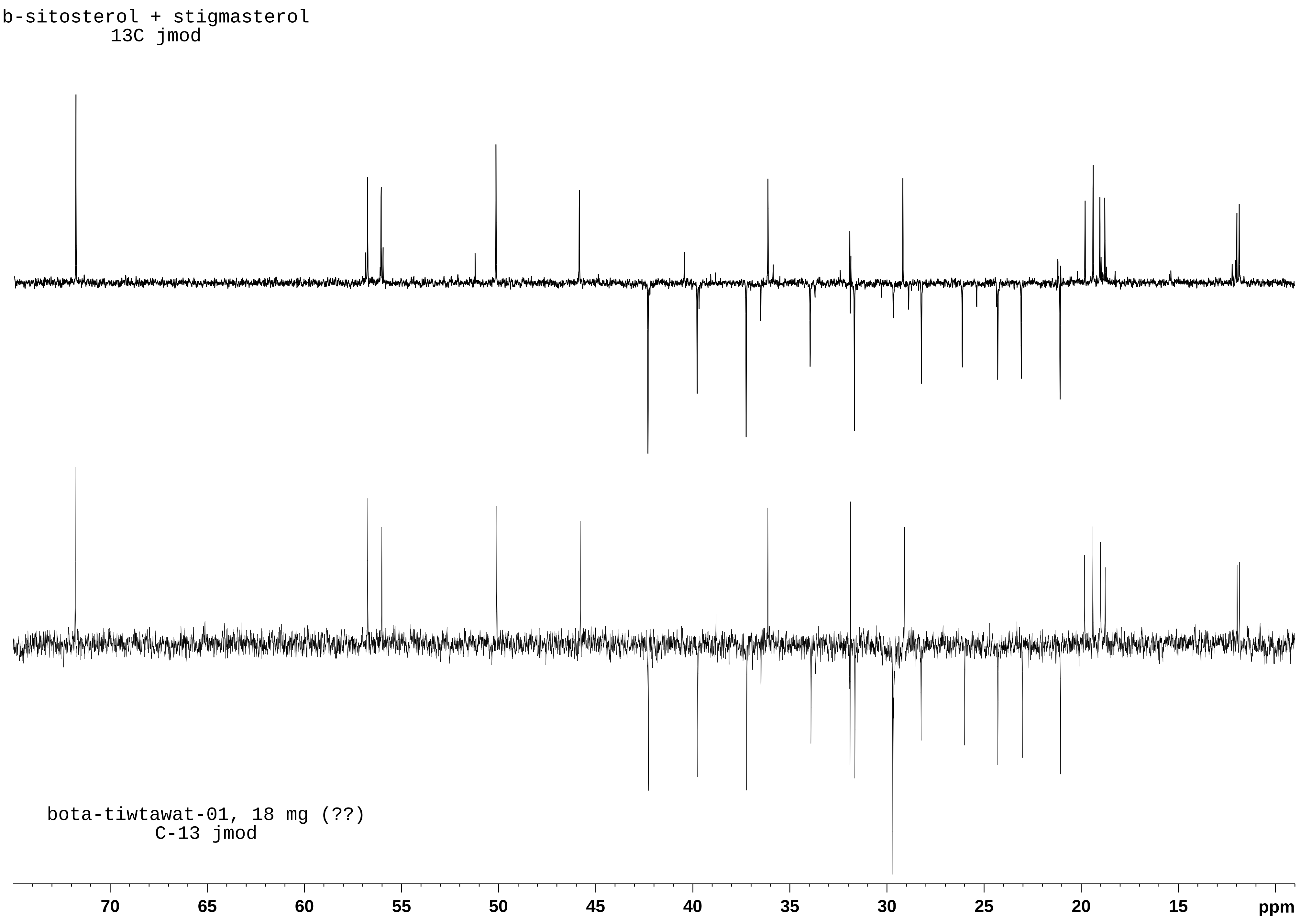
b-sitosterol + stigmasterol
13C jmod
bota-tiwtawat-01, 18 mg (??)
C-13 jmod
70
65
60
55
50
45
40
35
30
25
20
15
ppm
